# Corrections

**Published:** 1873-12

**Authors:** 


*Corrections.*—In the report of the proceedings of the Southern Dental Association, published in the November number of *Dental Cosmos*, on page 586, Dr. Gorgas is made to say that he framed the constitution of this Association; and also from what follows it would appear that Prof. Cutler had been recommended to fill a totally different position than the one suggested to the Association in the answer of the Faculty of the Baltimore College of Dental Surgery to the proposition made them. The following is a correct report of the remarks of Dr. Gorgas on this subject:

"Professor Gorgas, Dean of the Baltimore College, said that the proposition of the Association had been carefully considered and respectfully answered by the Faculty of the Baltimore College of Dental Surgery. He asked if the Association was able to assume the burden of supporting a College? Are the members willing to be subjected to a heavy tax in its behalf? Is any gentlemen present, able, and if able, willing to be subjected to a heavy annual tax to support any institution? The Southern Dental Association at the commencement of the present session, consisted of one hundred and twenty-seven members; and at least one hundred thousand dollars would be required to properly endow a dental college, in addition to all that could be received from students. As one of the founders of the Southern Dental Association, one of the committee who framed its constitution and by-laws, and its first Recording Secretary, he took great interest in its success, and would use his best efforts to have it succeed; therefore any proposition coming from this Association through him, would be zealously advocated.

"The Faculty of the Baltimore College of Dental Surgery placed it in the power of the Association to establish one professorship, and then by degrees to have control over the college by creating new professorships, or endowing old ones, as their means to do so from time to time justified. To place the Baltimore College in the hands of the Association at this time, would result in a failure to support it, and cause its downfall. He had no aspiration for office in the Association, was not a candidate for any office, and hence was honest in his endeavors to do justice to both the Association and the College he represented. He was surprised when he was elected to the office of 1st Vice-President at the Richmond meeting.

"He also stated that in his opinion, the objections against the proposition made to the Association by the Baltimore College, arose from the filling of the chair it was proposed to endow by the Faculty, thereby destroying the aspirations of some members of this Association who desire to become professors in a college, when they do not possess the qualifications necessary for such a position. Again, almost every one who complains of the colleges, is ignorant of their course of study and management, judging them by a few isolated cases occuring years ago when the country was convulsed by civil war, and the majority of the members of the Faculty absent in the army, and when a suspension would have forfeited their charter, and entailed the downfall of the institution by the persecution of the party then in power."

---

In an obituary notice of the late Dr. Amos Westcott, published in the *Missouri Dental Journal* the author states that the organization of the Baltimore College of Dental Surgery was due to the efforts of Dr. Westcott. While we admit that Dr. Westcott was a very efficient member of the Faculty of this institution in its early days, we think that great injustice has been done to others in making a statement of this nature which is so wide of the truth.

The founders of the Baltimore College of Dental Surgery, to whom a charter was granted in 1839 by the Legislature of the State of Maryland, were Drs. Chapin A. Harris, Horace H. Hayden, H. Willis Baxley, and Thos. E. Bond. Dr. Amos Westcott did not become a member of the Faculty of this institution until 1846, and resigned in 1849, as the records of the college, now in possession of the writer, will show.

This college therefore had been in existence for more than five years, and graduated five classes before Dr. Westcott became connected with it.

